# Comparative Evaluation of Effectiveness of IAVchip DNA Microarray in Influenza A Diagnosis

**DOI:** 10.1155/2014/620580

**Published:** 2014-11-23

**Authors:** K. T. Sultankulova, O. V. Chervyakova, N. S. Kozhabergenov, K. A. Shorayeva, V. M. Strochkov, M. B. Orynbayev, N. T. Sandybayev, A. R. Sansyzbay, A. V. Vasin

**Affiliations:** ^1^Research Institute for Biological Safety Problems (RIBSP), Science Committee of Ministry of Education and Science of Republic of Kazakhstan, Gvardeiskiy, Kordaiskiy Rayon, Zhambylskaya Oblast 080409, Kazakhstan; ^2^Research Institute of Influenza, Ministry of Healthcare of the Russian Federation, Prof. Popov Street 15/17, Saint Petersburg 197376, Russia

## Abstract

The paper describes comparative evaluation of IAVchip DNA microarray, reverse transcription PCR (RT-PCR), and real-time RT-PCR versus virus isolation in chicken embryos and shows their diagnostic effectiveness in detection and subtyping of influenza A virus. The tests were evaluated with use of 185 specimens from humans, animals, and birds. IAVchip DNA microarray demonstrates higher diagnostic effectiveness (99.45%) in early influenza A diagnosis as compared to the real-time PCR (98.38%) and RT-PCR (96.22%), thus showing its clear superiority. Diagnostic sensitivity of IAVchip DNA microarray (100%) exceeds the same of RT-PCR (95.95%) and real-time RT-PCR (97.96%) in the range of estimated confidence intervals. IAVchip DNA microarray and real-time RT-PCR displayed equal diagnostic specificity (98.85%), while diagnostic specificity of RT-PCR was 96.40%. IAVchip DNA microarray has an advantage over the other tests for influenza A diagnosis and virus identification as a more rapid method that allows performing simultaneous detection and subtyping of about tens of specimens within one experiment during 8–10 hours. The developed IAVchip DNA microarray is a general test tool that enables identifying simultaneously 16 hemagglutinin (HA) and 9 neuraminidase (NA) subtypes of influenza A virus and also to screen the influenza A viruses from humans, animals, and birds by M and NP genes.

## 1. Background

Influenza A virus (IAV) belongs to Orthomyxoviridae family. IAV is subtyped according to antigenic specificity of the surface glycoproteins hemagglutinin (HA) and neuraminidase (NA). Today 16 hemagglutinin and 9 neuraminidase subtypes are known [1]. Influenza A is contagious both for humans and animals. All IAV subtypes have been detected among water birds that are a natural IAV reservoir. The most frequent are 24 combinations of hemagglutinin and neuraminidase: H1N1, H2N2, H2N3, H3N2, H3N8, H4N2, H4N4, H4N6, H4N8, H5Nl, H5N2, H5N9, H6Nl, H6N2, H6N5, H6N9, H7N1, H7N2, H7N3, H7N7, H9N2, H9N8, H10N7, and Н11N9. Among horses influenza is caused by viruses of subtypes H7N7 and H3N8. Equine influenza virus of H3N8 subtype causes disease in dogs [2, 3]. Pigs are infected with influenza virus of subtypes H1N1, H1N2, H3N1, H3N2, and H2N3. The same viruses can affect humans. Only 3 HA subtypes (H1, H2, and H3) and 2 NA (N1, N2) have been detected among humans during the last century [4]. However cases of infection among humans with highly pathogenic IAV of subtypes such as H5N1, H7N7, H9N2, and H7N9 are recorded recently [5–8].

Owing to the high mutation rate of the surface HA and NA glycoproteins of IAV subtyping appears to be difficult; therefore, it is necessary to use supplementary methods and tools of diagnosis as well as their combinations to get reliable results [4, 8]. High pathogenicity of influenza viruses and great economic and social damage caused by the infection requires rapid and correct diagnosing.

So far, the DNA microarray-based detection is one of promising techniques in differential diagnosis of IAV infection, since it allows combining efficiency of the nucleic acid amplification and ample capacities of screening with use of biochip [9–14].

## 2. Objectives

IAVchip DNA microarray [15, 16] for early IAV detection and differentiation has been developed at the RIBSP (Research Institute for Biological Safety Problems) and now the ways of its use in practice are investigated. The objective of the study was to evaluate diagnostic effectiveness of IAVchip DNA microarray in differential detection of 16 hemagglutinin and 9 neuraminidase IAV subtypes as compared to virus isolation in chicken embryos, real-time PCR, and RT-PCR.

## 3. Study Design

### 3.1. Tested Samples

Smears and swabs from humans, animals, and birds (185 samples in total) were used as a test subject. Forty-two human samples were taken in Kazakhstanean hospitals under the Program of Epidemiological Surveillance in 2012 from cases suspected for acute respiratory viral infection and were kindly provided to our laboratory by the Republican sanitary-epidemiological station.

Eighty-five samples were collected in 2012 from horses with signs of a respiratory infection in Kostanaiskaya, Almatinskaya, and Zhambylskaya oblasts under the Republican Science-and-Technology Program “Epizootiological monitoring of the territory of Kazakhstan, Central Asian and Neighboring Countries for Equine Influenza”.

Fifty-eight samples from wild birds were taken in 2005-2006 and in 2012 in Akmolinskaya and Karagandinskaya oblasts under the Program of Avian Influenza Surveillance.

Sampling was carried out following WHO recommendations [17]. Nasal smears (swabs) were taken from humans and horses using dry sterile probes with cotton tips; cloacal swabs were taken from birds in the same way. After sampling the cotton pellet (working part of the probe) was placed into a cryotube with sterile transport medium (transport media consisted of Hanks balanced salt solution supplemented with 10% glycerol, 200 U/mL penicillin, 200 mg/mL streptomycin, 100 U/mL polymyxin B sulphate, 250 mg/mL gentamicin, and 50 U/mL nystatin). Cryotubes with samples were transported in liquid nitrogen (−196°C).

### 3.2. Control Samples

One has the following:

- plasmid DNA containing IAV M gene;
- plasmid DNA containing IAV NP gene.

M gene (EU213070.2) and NP gene (EU213048.1) were cloned into strain XL1-Blue* E. coli* using pGEM-T VectorSystems (Promega). After that plasmid DNAs containing M and NP genes of influenza A virus were extracted using QIAprep Spin Miniprep Kit (Qiagen).

### 3.3. Assay of Samples with Use of IAVchip DNA Microarray for Influenza A Diagnosis and Virus Subtyping

#### 3.3.1. RNA Extraction

RNAs were extracted from samples with the help of TRizol (“Invitrogen,” USA) following the manufacturer's instructions.

#### 3.3.2. Viral RNA Amplification

One-step RT-PCR was performed with use of Super Script III Platinum One-Step Quantitative RT-PCR System (“Invitrogen,” USA) following the manufacturer's instructions. The universal primer pair selected for the terminal highly conservative regions that are present in all IAV genome segments (MBTuni-12 and MBTuni-13) has been used in multisegment amplification [18]. Conditions of RT-PCR are as follows: SuperScriptIII RT/PlatinumR Taq Mix—1 *μ*L; 2X Reaction Mix—25 *μ*L; primers 10 *μ*M—1.4 *μ*L each; RNaseOUT—1 *μ*L; RNA (1 pg to 1 *μ*g)—10 *μ*L; DEPC-treated water up to 50 *μ*L [15]. Fluorescent labeling was made via direct integration of Cy5-dCTP (“DNA-Synthesis,” Russia) immediately in the process of RT-PCR and the reaction mixture contained extra 33 *μ*M of 1 mM Cy5-dCTP.

#### 3.3.3. Assay with Use of IAVchip DNA Microarray

**Figure 1:**
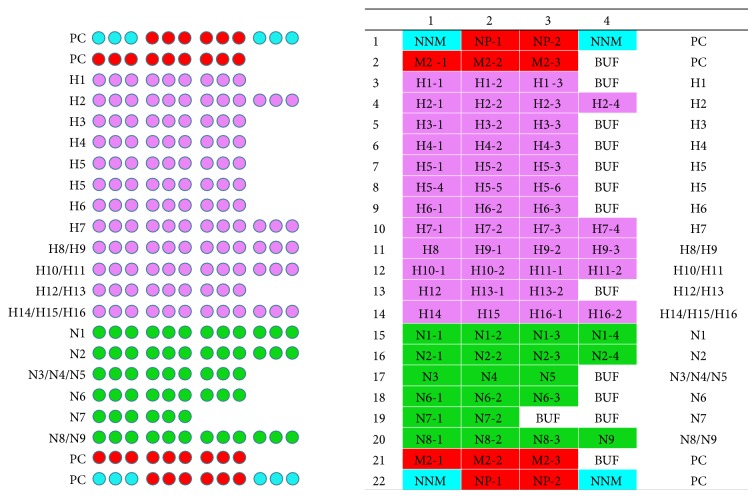
Scheme of the model IAVchip microarray for influenza A virus subtyping. H: hemagglutinin, N: neuraminidase, PC: positive control, BUF: 1xMicroarray printing buffer Arrayit, and NNM: hybridization control.

An experimental batch of microarrays was printed on aldehyde substrate (Vantage Aldehyde Slides “CEL Associates”) by the method of contact printing of oligonucleotide probes in Nano Print LM 60 (“Arrayit,” USA) in accordance with the scheme on Figure 1.

The scheme includes the probes corresponding to hemagglutinins HA1–HA16 and then to neuraminidases NA1–NA9. In addition there is a universal oligonucleotide probe to the region of IAV genes that encode M and NP proteins shared by all IAV subtypes. Oligonucleotide probes used in the experiments are shown in Table 1.

One microarray slide allows simultaneous analysis of 16 different IAV strains and isolates.

IAVchip DNA microarray was validated with use of IAV reference strains of different subtypes, origin, and biological characteristics [16].

#### 3.3.4. Hybridization

To 1 *μ*L of PCR-mixture containing Cy5-cDNA hybridization solution was added; the total volume was brought with H_2_O up to 50 *μ*L and heated in the solid-state thermostat at 99°C for 2 min and then cooled in ice for 2 min and at once applied onto the microarray. In parallel the oligonucleotide probes on the microarray were denatured by boiling of the slide in H_2_O for 1 min followed by incubation in 96% ethanol (C −20°C) for 1 min. After that the slide was dried by centrifugation at 300 g for 2 min. Hybridization was performed with use of a frame for 16 subarrays FAST Frame (“Whatman”, USA) for 2 h at 37°C and stirring at 250 rpm. After hybridization the slide was rinsed in 3×SSC buffer for 2 min and in 1×SSC buffer for 2 min to remove unbound molecules of the sample and hybridization buffer. After that the frame was removed and the slide was rinsed with water for 2 min. It was dried by centrifugation at 300 g for 2 min.

#### 3.3.5. Scanning

The microarrays were scanned in InnoScan710AL (“Innopsys,” France) with 5 *μ*m resolution. Fluorescent scanning was performed at wavelengths 532 nm and 635 nm. The resulted images were processed with use of the software Mapixver. 5.5.0 (“Innopsys”, France).

### 3.4. Virus Isolation in Chicken Embryos

Virus isolation was performed in 9-10-day chicken embryos (CE) following the standard procedures where influenza virus production was confirmed by identification in hemagglutination inhibition (HI) test [19].

### 3.5. Real-Time RT-PCR

Real-time RT-PCR was performed by method of “TaqMan” with use of Light Cycler 2.0 manufactured by Roche Company. Real-time RT-PCR for detection of Н1N1 and Н3N2 subtypes was performed using primers and conditions described by Schweiger et al. (2000) [20], for detection of Н3N8 using primers and conditions described by Lu et al.  (2009) [21] and for detection of Н5N1 using primers and conditions according to WHO guidelines (2007) [22].

### 3.6. RT-PCR

RT-PCR for detection of Н1N1 and Н3N2 subtypes was performed according to Schweiger et al.  (2000) [20], for detection of Н3N8 according to Lu et al.  (2009) and Chervyakova et al.  (2014) [21, 23], and for detection of Н5N1 subtype according to WHO guidelines (2007) [22].

Specific regions of IAV cDNA were produced in GeneAmp PCR 9700, Applied Biosystems.  Table 2 shows oligonucleotide primers that were used in the study.

### 3.7. Statistical Analysis

True positive (TP), true negative (TN), false positive (FP), and false negative (FN) results of the assays were used to assess effectiveness of laboratory tests.

The following calculations were used: sensitivity  (SN) = (TP/TP + FN), specificity (SP) = (TN/TN + FP), Positive Predictive Value  (PPV) = (TP/TP + FP), Negative Predictive Value  (NPV) = (TN/TN + FN), and diagnostic effectiveness  (DE) = (TP + TN/TP + FP + FN + TN)  [24].

95% confidence intervals (95% CI) were calculated according to van Engelsdorp et al. [24].

## 4. Results

### 4.1. Detection of Influenza A Virus in Clinical Specimens

Diagnostic effectiveness of IAVchip DNA microarray in comparison to the virus isolation in chicken embryos, real-time RT-PCR, and RT-PCR for IAV detection and subtyping was evaluated by testing 185 clinical specimens from humans, animals, and birds.

“True” state of infection is determined by the most accurate diagnostic method that is called “gold standard.” In diagnostics and identification of the influenza infection virus isolation in chicken embryos followed by identification in HI is the “gold standard” [19].

Table 3 shows the results of detecting IAV in clinical specimens by various methods in comparison to virus isolation in chicken embryos.

In our experiments IAV was isolated by the method of virus isolation in chicken embryos from 98 (52.97%) specimens out of 185 clinical samples taken from humans, animals, and birds. The rest of samples (87, i.e., 47.03%) showed negative result. The same picture was observed in the experiments aimed at IAV isolation with use of IAVchip DNA microarray. In these tests the influenza A virus was detected in 98 (52.97%) samples. At the same time the method of real-time PCR displayed the presence of IAV only in 96 (51.89%) specimens, and the method of RT-PCR merely in 71 (38.38%) samples. Percentage of false positive results in the tests with use of IAVchip DNA microarray and in the real-time PCR was 0.54% and 2.16% in RT-PCR. Use of IAVchip DNA microarray did not show false negative responses, while real-time PCR and RT-PCR displayed 1.08% and 1.62%, respectively.

### 4.2. Comparison of Various Tests for Influenza A Diagnosis

Sensitivity and specificity of each test were determined to evaluate effectiveness and reliability of influenza A diagnostic tests.

Sensitivity and specificity indices, as well as positive and negative prognostic values of the developed IAVchip DNA microarray, of RT-PCR and real-time RT-PCR with 95% confidence interval are displayed in Table 4.

Method of virus isolation in chicken embryos being used as a standard, IAVchip DNA microarray displayed 100% diagnostic sensitivity and 98.85% diagnostic specificity within the range 97.35–100% of 95% CI. Diagnostic sensitivity of IAVchip DNA microarray (100%) exceeds the same parameter of real-time RT-PCR (97.96%) within the range 95.96%–99.96% of 95% CI and of RT-PCR (95.95%) within the range 93.15%–98.75% of 95% CI.

In influenza A diagnosis IAVchip DNA microarray and real-time PCR demonstrated equal diagnostic specificity (98.85%), while the same characteristic of RT-PCR was 96.40%. Diagnostic specificity of IAVchip DNA microarray and real-time PCR were in the range of the confidence intervals of the RT-PCR specificity. The limit of the 95% confidence intervals for RT-PCR was 93.70%–99.10%.

PPV and NPV values of IAVchip DNA microarray were 98.99% and 100%, respectively. PPV and NPV of the real-time RT-PCR were also in the range of the estimated confidence intervals.

### 4.3. Influenza A Virus Subtyping with Use of IAVchip DNA Microarray

IAVchip DNA microarray, real-time RT-PCR, and RT-PCR were used to subtype IAV from 98 samples proved to be positive by the method of the virus isolation in chicken embryos. The IAV were subtyped by the methods of RT-PCR and real-time RT-PCR in 3 steps: identification of the virus (step 1), detection of HA (step 2), and NA (step 3). IAVchip DNA microarray was used to subtype simultaneously the tested IAV with probes specific to hemagglutinin 1–16 and neuraminidase 1–9 genes.  Table 5 shows examples of IAV subtyping with the help of IAVchip DNA microarray in the form of histograms.

As Table 5 shows IAVchip DNA microarray makes possible simultaneous IAV subtyping in the tested samples. Possibility in principle to use the DNA microarray for diagnosis and subtyping of various influenza viruses has been demonstrated by examples of seasonal A/H1N1 and A/H3N2 viruses, equine influenza A/H3N8 virus, and highly pathogenic avian influenza A/H5N1 virus. In the assay of the samples the value of specific fluorescence reliably exceeded the value of the background fluorescence (*P* < 0.05). Moreover, M and NP genes were reliably detected in all samples.

The results of comparative analysis of samples with use of IAVchip DNA microarray, as well as by real-time RT-PCR and RT-PCR, are shown in Table 6.

The findings of the study showed that by use of IAVchip DNA microarray influenza viruses were detected in all 98 samples; 36.73% of them were subtyped as influenza A virus A/Н1N1, 40.82% as A/H3N2, 14.29% as A/H3N8, and 8.16% as A/H5N1.

In the same samples influenza viruses were detected and subtyped by RT-PCR as follows: in 24.49% of samples as A/Н1N1, in 31.63% as A/H3N2, in 11.22% as A/H3N8, and in 5.10% as A/H5N1. The results of RT-PCR were positive in 72.45% samples; in 27.55% they were negative.

In the same 98 samples real-time RT-PCR detected and subtyped the following influenza viruses: A/Н1N1 in 35.71%, A/H3N2 in 39.80%, A/H3N8 in 14.29%, and A/H5N1 in 8.16% of samples. Real-time RT-PCR diagnosed influenza A virus in 97.96% of all assayed samples and showed negative result in 2.04% of samples.

## 5. Discussion

IAV genome variability begets a very important diagnostic problem that consists in need of rapid and accurate method of diagnosis not only of existing but of emerging viruses for efficient influenza surveillance at the global level [25, 26].

Biological microarrays are developed today in many countries. Biochips for diagnosis of the human influenza of subtypes H1N1, H3N2, including H5N1, are set forth [27]. It should be noted that various approaches were used. MChip was developed with use of probes only to M gene of the influenza virus, but further on it was supplemented with probes to hemagglutinin and neuraminidase genes (FluChip) [11]. The microarrays proposed by Huang et al. [1] and Teo et al. [28] contain probes to the hemagglutinin and nonstructural protein genes of the influenza B virus. These diagnostic microarrays can be used only for diagnosis of the seasonal influenza because reassortment and mutations so characteristic of the influenza virus result in emergence of new subtypes that are isolated both from animal and bird populations and from humans.

There are also microarrays for identification of all IAV subtypes by both hemagglutinin and neuraminidase genes. Xueqing et al. [29] used 52 oligonucleotide probes for typing IAV. The viral cDNA was amplified in multiplex PCR with 25 pairs of primers. Multiplex PCR for amplification of the influenza virus cDNA with use of 25 pairs of primers was performed by Han et al. [14]. The component structure was optimized for performance of the reaction in 4 tubes. Quan et al. [30] developed microarray GreeneChipResp that enables identifying 20 respiratory viral infections apart from typing all subtypes of IAV.

On the basis of the oligonucleotide microarray IAVchip that allows detecting and subtyping IAV has been developed at the RIBSP [15]. This microarray is universal for all subtypes of IAV and when necessary enables screening the IAV not only by HA and NA, but by M and NP genes as well.

Diagnostic effectiveness (DE) of the test expressed as percentage ratio of the number of true test results to the total number of findings for IAVchip DNA microarray was 99.45%, for the real-time PCR and RT-PCR; it was 98.38% and 96.22%, respectively.

The majority of authors propose in their papers using microarrays for the human influenza viruses [9, 11, 31], whereas our biological chip is designed to identify influenza viruses from humans animals and birds.

Analysis of these data shows that IAVchip DNA microarray for IAV diagnosis and identification has the advantage over the other tests in effectiveness and is more rapid owing to possibility to detect and subtype several influenza A viruses simultaneously in one experiment.

It should be noted that in the course of testing clinical specimens with use of IAVchip DNA microarray in one reaction both influenza A virus identification and subtyping of tens of viruses take place during 8–10 hours. Subtype information is especially important, for example, in the South-Eastern Asia, where subtypes A/H3N2, A/H1N1, and A/H5N1 can simultaneously circulate in a body.

So, possible usage of the microarray in clinical practice for influenza A diagnosis and subtyping of the viruses isolated both from humans and from animals and birds is shown.

## Figures and Tables

**Table 1:** Oligonucleotide probes for influenza A virus subtyping.

Subtype	Probe	Sequence (5′ → 3′)^*^
hemagglutinin		
1	H1-1	gaagggagaatgaactattactggacactagtagagccgggagacaa
	H1-2	gtctccctgggggcaatcagyttctggatgtgytcyaatggg
	H1-3	acaggactaaggaacatcccatccattcaatccagaggtttgtttgg

2	H2-1	taaggaatgttccccagattgaatcaagaggattgtttggggcaat
	H2-2	gtcaccgtgactcatgccaaggacattcttgagaaaacgcataatgg
	H2-3	tatgctacagtagcaggytccctgtcactggcaatcatgata
	H2-4	tgggatgtcataaattttgagagcactggtaatttaattgcaccagaata

3	H3-1	atgtgggcctgccararaggcaacattaggtgcaacatttgc
	H3-2	atgtgggcttgccaaaaaggcaacatcagatgcaacatttgc
	H3-3	cagcaactgttacccttatgatgtgccggattatgyctccct

4	H4-1	gcactrcttttagcctttattttgtgggcttgtcagaatggaaacat
	H4-2	attttgtgggcttgtcagaayggaaacatccggtgccagatttg
	H4-3	ttccatatcatgcttyttgctcgttgcactrcttttagcctt

5	H5-1	ttgggacatcaacactaaaccagagattggtaccaaraatagctactaga
	H5-2	ttattcaacagtggcragttccctagcactggcaatcatggt
	H5-3	cccaacaataaagagragytacaataataccaaccaagaagatcttttgg
	H5-4	cccaacaataaagaggacctayaacaacaccaatgtagaagaccttttaa
	H5-5	tttatagagggaggatggcagggaatggtagatggttggtatgg
	H5-6	ctagatgtctggacttataatgctgaacttctggttctcatggaaaatga

6	H6-1	cttggtgtgtatcaaattcttgcyatttatagtacggtatcgagcag
	H6-2	caaatccttgcyatttatagtacggtatcgagcagtctrgttttgg
	H6-3	gcaatgggtctttggatgtgttcaaatggttcaatgcartgca

7	H7-1	atgggattggttttcatttgcataaagaatggaaacatgcrgtgcactat
	H7-2	cttcggggcatcatgtttcatacttctggccattgcaatggg
	H7-3	catcaaaatgcacaaggagarggaactgcagctgactacaaa
	H7-4	tggtttagcttcggggcatcatgcttcctwcttcttgccattgcaatggg

8	H8	atttacagtacagtggcggccagtctytgcttggcaatcctg

9	H9-1	cttacaaaatcctyaccatttattcgactgtcgcctcatctcttgt
	H9-2	gcaatggggtttgctgccttcytrttctgggccatgtccaat
	H9-3	ttctgggccatgtcyaatggatcttgcagatgcaacatttgtat

10	H10-1	cttttggctgtcatcatggggcttgttttcttctgtytgaaaaatggaaa
	H10-2	gtcatcaattggacyaaggattcaataaccgacatctggacttatcarg

11	H11-1	gatctccatgattctaatgttcgaaacctccatgaaaaggtcagacgaat
	H11-2	tgggcgtgcagyaatggatcatgtagatgtaccatttgcatt

12	H12	tactgctcatgattattgggggtttcattttcggrtgtcaaaatggaaat

13	H13-1	agtgttgtgytagtaggactcatactctctttcatcatgtgggcc
	H13-2	ataaatatgcttgcagacagaatagatgaygctgtaactgatgta

14	H14	tgcatcacccatcaagcgataatgagcaaacggatctctacaagg

15	H15	gctgatctgataatagaaagaagaaattcaagtgacatctgttacccagg

16	H16-1	taatgccattgatgaaggagatggttgcttcaatcttcttcacaa
	H16-2	attcgaaatgggacatataatcatgaggactacaaagaagagtcacaa

Neuraminidase		
1	N1-1	gggttggtcttggccagacggtgctgagttgccattyaccatt
	N1-2	tcctaatggatggacarataccgacagtgatttctcagtgaaacaggatg
	N1-3	tggtcttggccagacggtgctgagttgccvttcaccattgac
	N1-4	caagagtctgaatgtgcatgtgtaaatggytcttgctttactgtaatgac

2	N2-1	caagtgtgyatagcatggtccagctcaagttgtcacgatggaaa
	N2-2	tttggsraccaaacaagtgtgcatagcatggtccagctcaag
	N2-3	ttttgtggcacttcaggyachtatggaacaggctcatggcct
	N2-4	tgtgghacytcaggtacatatggaacaggctcatggcctgat

3	N3	agyaatagtatagttactttctgtggaytagacaatgaacctggatcggg

4	N4	tgtggtgttaattctgataccacaggttggtcatggccygatggc

5	N5	ttttgtggtgtttcmagtgaggtcccaggrtggtcctgggatgatgg

6	N6-1	tcatgccatgacggcatctcaagaatgtcratctgcatgtca
	N6-2	gagcgattrggatcatggtcctggcatgatggtgctgaratc
	N6-3	gagcgattgggatcktggtcatggcatgatggggctgaaatc

7	N7-1	gttgaaggatgggtagtggtggcyaaggacaatgccataagatt
	N7-2	cagttgggtccggttccttccccgatggggcacaratccaat

8	N8-1	atatggacctcwagyagctccattgtgatgtgtggagtagaycat
	N8-2	tgagtgtagaaatagggcaatcacccaatgtgtaccaggcaaggt
	N8-3	tggtcrtggcacgatggagctattcttccytttgacatcgat

9	N9	atgtgttccagyacagaattcctkggacaatggaactggcctgat

M2-protein		
	M2-1	gcagartgctgtggatgttgacgatrgtcattttgtcaacatag
	M2-2	cctatcagaaacgaatgggggtgcagatgcaacgattcaagtga
	M2-3	ccttctacggaaggagtrccwgagtctatgagggaagaatatcg

NP protein		
	NP-1	acgaaaaggcaacgaacccgatcgtgccttcctttgacatga
	NP-2	atgagtaatgaaggdtcttatttcttcggagacaatgcagargag

**Table 2:** Oligonucleotide primers and probes for influenza A virus typing and subtyping in real-time PCR and RT-PCR.

Influenza virus type/subtype	Primer or probes	Sequence
A	AM-151	CATGGAATGGCTAAAGACAAGACC
	AM-397	AAGTGCACCAGCAGAATAACTGAG
	Probe AM-245	CTGCAGCGTAGACGCTTTGTCCAAAATG
A/H1N1		
A/H1	HA1-583	GGTGTTCATCACCCGTCTAACAT
	HA1-895	GTGTTTGACACTTCGCGTCACAT
	Probe HA1-783	TGCCTCAAATATTATTGTGTCCCCGGGT
A/N1	NA1-1078	ATGGTAATGGTGTTTGGATAGGAAG
	NA1-1352	AATGCTGCTCCCACTAGTCCAG
	Probe NA1-1138	TGATTTGGGATCCTAATGGATGGACAG
A/H3N2		
A/H3	HA3-115	GCTACTGAGCTGGTTCAGAGTTC
	HA3-375	GAAGTCTTCATTGATAAACTCCAG
	Probe HA3-208	CTATTGGGAGACCCTCATTGTGATGG
A/N2	NA2-560	AAGCATGGCTGCATGTTTGTG
	NA2-858	ACCAGGATATCGAGGATAACAGGA
	Probe NA2-821	TGCTGAGCACTTCCTGACAATGGGCT
A/H3N8		
A/H3	EqFlu HA3 F	TCACATGGACAGGTGTCACTCA
	EqFlu HA3 R	GGCTGATCCCCTTTTGCA
	EqFlu HA3 Pr	AACGGAAGAAGTGGAGC
A/N8	N8Eq-F30	TGG ATC TGC ATC ATT GGG GA
	N8eq-R535	CTG ACC ATG CCA CCG ATT CA
A/H5N1		
A/H5	H5HA-205-227v2-F	CGATCTAGAYGGGGTGAARCCTC
	H5HA-326-302v2-R	CCTTCTCCACTATGTANGACCATTC
	H5-Probe-239-RVa	FAM-AGCCAYCCAGCTACRCTACA-MGB
	H5-Probe-239-RVb	FAM-AGCCATCCCGCAACACTACA-MGB
A/N1	N1-For-474-502	TAYAACTCAAGGTTTGAGTCTGTYGCTTG
	N1-Rev-603-631	ATGTTRTTCCTCCAACTCTTGATRGTGTC
	N1-Probe-501-525	FAM-TCAGCRAGTGCYTGCCATGATGGCAMGB

AM: M gene of influenza A viruses.

HA1, HA3, and HA5: HA gene of influenza A viruses of subtypes H1, H3, and H5, respectively.

NA, NA2, and NA8: NA gene of influenza A viruses of subtypes N1, N2, and N8, respectively.

**Table 3:** Comparison of the results of our test using IAVchip DNA-microarray with the results of real-time RT-PCR and RT-PCR.

Result	Test			
	RT-PCR	Real-time PCR	IAVchip DNA microarray	Virus isolation in chicken embryos
Positive	71 (38.38%)	96 (51.89%)	98 (52.97%)	98 (52.97%)
False positive	4 (2.16%)	1 (0.54%)	1 (0.54%)	0
False negative	3 (1.62%)	2 (1.08%)	0	0
Negative	107 (57.84%)	86 (46.49%)	86 (46.49%)	87 (47.03%)

Total	185 (100%)	185 (100%)	185 (100%)	185 (100%)

**Table 4:** Comparative diagnostic value of different tests in influenza A diagnosis.

Result, %	Test		
	RT-PCR	Real-time RT-PCR	IAVchip DNA microarray
SN, 95% CI	95.95 (93.15–98.75)	97.96 (95.96–99.96)	100
SP, 95% CI	96.40 (93.70–99.10)	98.85 (97.35–100)	98.85 (97.35–100)
PPV, 95% CI	94.67 (91.37–97.97)	98.97 (97.57–100)	98.99 (97.59–100)
NPV, 95% CI	97.27 (94.97–99.57)	97.73 (95.63–99.83)	100

SN: sensitivity; SP: specificity; PPV: Positive Predictive Value; NPV: Negative Predictive Value; 95% CI: 95% confidence interval.

**Table 5:** Results of influenza A virus subtyping with use of IAVchip DNA microarray.

Subtypes	A	B
H1N1	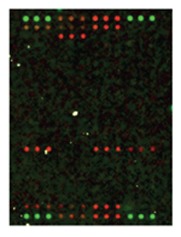	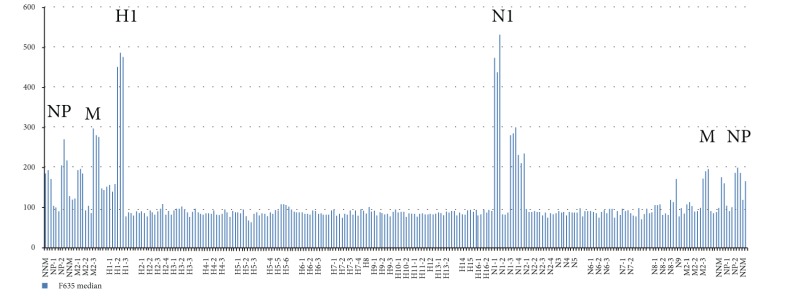

H3N2	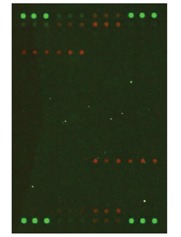	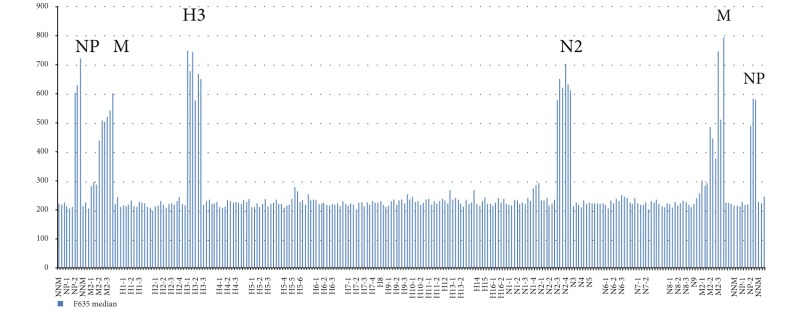

H3N8	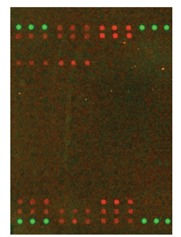	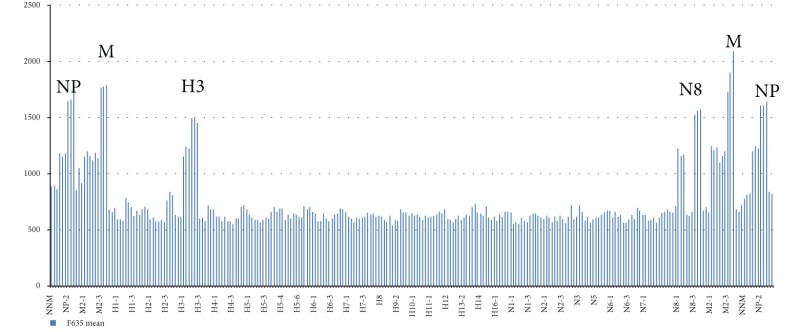

H5N1	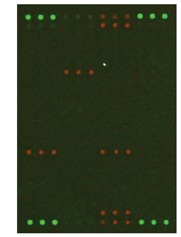	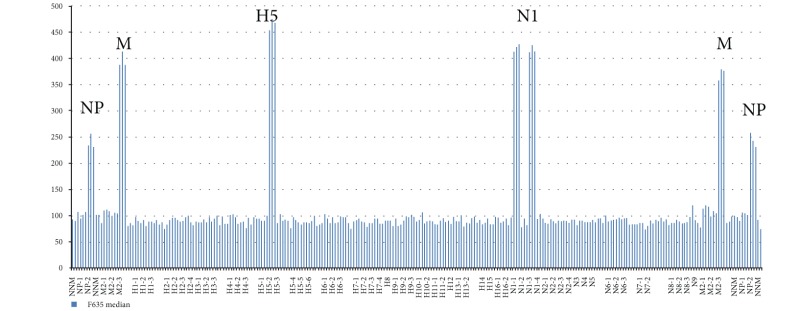

A: hybridization pattern of the assayed influenza virus.

B: results of subtyping after mean value counting of fluorescent spots; H1, H3, and H5: hemagglutinin gene subtypes; N1, N2, and N8: neuraminidase gene subtypes; M, NP: markers of nucleoprotein and matrix protein genes.

**Table 6:** Results of detecting influenza A virus subtypes in clinical specimens by various tests.

Subtype	Test						
	Reverse transcription PCR			Real-time RT-PCR			IAVchip DNA microarray
	Step 1	Step 2	Step 3	Step 1	Step 2	Step 3	Step 1
	Type A	H	N	Type A	H	N	Type A, H, N
Н1N1	24 (24.49%)	24 (24.49%)	24 (24.49%)	35 (35.71%)	35 (35.71%)	35 (35.71%)	36 (36.73%)
H3N2	31 (31.63%)	31 (31.63%)	31 (31.63%)	39 (39.80%)	39 (39.80%)	39 (39.80%)	40 (40.82%)
H3N8	11 (11.22%)	11 (11.22%)	11 (11.22%)	14 (14.29%)	14 (14.29%)	N/A	14 (14.29%)
H5N1	5 (5.10%)	5 (5.10%)	5 (5.10%)	8 (8.16%)	8 (8.16%)	8 (8.16%)	8 (8.16%)
Positive	71 (72.45%)	71 (72.45%)	71 (72.45%)	96 (97.96%)	96 (97.96%)	82 (83.67%)	98 (100%)

Note: 98 specimens were assayed.

## Abbreviations

IAV: Influenza A virus
HA: Hemagglutinin
NA: Neuraminidase
TP: True positive
TN: True negative
FP: False positive
FN: False negative
SN: Sensitivity
SP: Specificity
PPV: Positive Predictive Value
NPV: Negative Predictive Value
DE: Diagnostic effectiveness
95% CI: 95% confidence intervals.
